# Immunocompetence in adults: more than HIV negative

**Published:** 2016-09-30

**Authors:** Andres Felipe Zea-Vera

**Affiliations:** Departamento de Microbiología, Facultad de Salud, Universidad del Valle. Cali, Colombia

Dear editor

I read a case report about Tuberculosis and fungal co-infection in a previously healthy patient published in Colomb Med (Cali) by Fontalvo *et al*. 1 ( https://www.ncbi.nlm.nih.gov/pmc/articles/PMC4975131/), and I would like to address some related comments. Frequently clinicians report adult cases of patients with opportunistic infections as disseminated tuberculosis and/or fungal infections in patients consider as immunocompetent based mainly in the absence of human immunodeficiency virus infection (HIV negative). Immunocompetence is more complex than absence of HIV infection and involves a normal capacity to develop an immune response following the exposure to an antigen or broadly a normal immune response, but usually immunocompetent is define as the opposite of immunodeficiency. In the report authors said "Our aim is to report the case of an immunocompetent patient diagnosed with Mycobacterium tuberculosis and *Candida albicans* co-infections" but my deliberation is Do we make in the clinical practice all the efforts to consider a patient as immunocompetent?

Mycobacterial, fungal and other opportunistic infections force the clinician to rule out a large list of conditions associated with secondary immunodeficiency as infectious agents (HIV, Herpesvirus, HTLV), drugs (steroids, immunosuppressants, biologics, chemotherapy) , metabolic diseases (diabetes, renal failure, cirrhosis), malignancies (leukemia, lymphomas and solid tumors) and environmental conditions (radiation, heavy metals) 2 but even adult patients can have late onset primary genetic immunodeficiency disorders as chronic granulomatous disease, X-linked agammaglobulinemia, interleukin-12 receptor deficiency or interferon-gamma (IFN-γ) and interleukin-23/interleukin-17 pathway defects 3 explaining their pattern of infection or the presence of opportunistic microorganism. When a patient with opportunistic infections is assessed cellular immune response evaluation is mandatory, not only CD4+ and CD8+ T lymphocytes absolute quantification (not evaluated in this case report) but also qualitative T cell responses (v.g lymphoproliferation, cytokine production) as well as B cell and natural killer (NK) cells evaluation. Opportunistic infections in adult patients can also be a presentation of autoantibodies that inhibit cytokines including (but not only) anti interferon-gamma (anti IFN-γ) self-antibodies in previously healthy adults presenting with severe Mycobacterial infections  4 or antibodies to interleukin-17 (IL-17) and IL-22 that are associated with chronic candidiasis 5 this group of autoantibodies are now recognized as phenocopies or acquire immune disorders resembling primary genetic immunodeficiency diseases 6. From my point of view the term immunocompetent should be use more carefully.

Interestingly the patient presented had mild macrocytic anemia (hemoglobin 10.7 g/dL and mean corpuscular volume 103 μm^3)^. This feature is found frequently in patients with anti-cytokines autoantibodies and is related with self-antibodies to gastric parietal cell and to intrinsic factor producing pernicious anemia 7. Patients with adult onset immunodeficiency due to anti IFN-γ autoantibodies could be more susceptible to autoimmune disorders, requiring a higher suspicious index. The intent of this letter is to generate a wake-up call for better evaluation of patients with recurrent or opportunistic infections.
